# Case report: Therapeutic use of bortezomib in a patient with Schnitzler syndrome

**DOI:** 10.3389/fimmu.2025.1520470

**Published:** 2025-01-28

**Authors:** Hua Bai, Dongming Zhou, Jinwen Liu, Jie He, Zhou Min, Wenyong Fan, Bing Chen, Yong Xu

**Affiliations:** ^1^ Department of Hematology, Nanjing Drum Tower Hospital, Affiliated Hospital of Medical School, Nanjing University, Nanjing, China; ^2^ Department of Orthopedics, Tongji Hospital affiliated to Tongji University School of Medicine, Tongji University, Shanghai, China

**Keywords:** Schnitzler syndrome, IgM monoclonal gammopathy, plasma cell, urticarial rash, inflammatory response, bortezomib

## Abstract

Schnitzler syndrome (SchS) is a rare acquired systemic autoinflammatory disorder, characterized by chronic urticarial rash and immunoglobulin M (IgM) monoclonal gammopathy. Anti-interleukin-1 (IL-1) therapies have been shown to be more effective in managing the clinical symptoms of SchS compared to anti-IL-6 therapies. In this case report, we present a male patient with urticarial rash, fever, and arthralgia. Laboratory tests identified the presence of IgMκ monoclonal protein, and the absence of IL-1β in serum. Whole exome sequencing (WES) did not reveal any pathological variants associated with monogenic autoinflammatory diseases or the *MYD88* L265P mutation. He met the diagnostic criteria for SchS and was treated with bortezomib, leading to a significant improvement in clinical symptoms and a decline in IgMκ monoclonal protein levels. The patient tolerated the treatment well. This case suggests that bortezomib may be considered as a potential treatment option for SchS, in addition to anti-IL-1 therapies and bruton tyrosine kinase (BTK) inhibitors.

## Introduction

Schnitzler syndrome (SchS) is an adult-onset, rare, autoinflammatory disorder characterized by urticarial rash and monoclonal gammopathy (usually of the immunoglobulin M, IgM, rarely IgG). This disease usually manifests around the age of 50 and is clinically marked by urticarial rash, fever, arthralgia, lymphadenopathy, and elevated inflammatory markers (1, 2). The central pathogenesis of SchS is dysregulation of interleukin (IL)-1β and the inflammasome pathway. The long-term follow-up of IL-1 blockade in SchS demonstrates that canakinumab treatment effectively reduces clinical signs and symptoms, decreases inflammatory markers, and improves quality of life over a four-year period (3). However, as observed in a comprehensive study of 281 cases of SchS, the efficacy of anti-IL-1 or anti-IL-6 therapies may decrease over time in some patients, underscoring the importance of continuous monitoring and possible modifications to the therapeutic approach (2).

While the rarity and complexity of SchS can make its initial diagnosis challenging and sometimes delayed, 20% of SchS patients often have lymphoproliferative disorders, including multiple myeloma (MM) and lymphoplasmacytic lymphoma (LPL), and its specific variant, Waldenstrom's macroglobulinemia (WM), which is the most frequent form of LPL (4). Moreover, IL-1 or IL-6 targeting therapy cannot prevent the occurrence of lymphoproliferative diseases. Current evidence indicates that while these treatments can control symptoms and reduce inflammation, they may not alter the underlying monoclonal gammopathy or plasma cell dyscrasia, which are associated with the development of such disorders (4, 5). So we need to explore new therapeutic drugs. MYD88 is at the crossroad of toll-like receptor, IL-1R, and B-cell receptor pathways and may participate in uncontrolled inflammation. A key pathogenic factor is the *MYD88* L265P gain-of-function mutation, which promotes cell survival by forming a protein complex of IL-1 receptor-associated kinase 1 (IRAK1) and IRAK4, contributing to up-regulation of IL-1 signaling and other pro-inflammatory pathways (6). BTK is involved in toll-like receptor signaling, which also involves the adaptor protein MYD88. The activating *MYD88* L265P mutation causes MYD88 to spontaneously assemble protein complexes that trigger pro-survival signaling along multiple pathways (7, 8). Considering the obvious specificity of *MYD88* L265P mutation for inflammasome activation, and the occurrence of this mutation in WM, IgM monoclonal gammopathy of undetermined significance (MGUS) and SchS patients. Treatment with bruton tyrosine kinase (BTK) inhibitor ibrutinib has proven effective in two cases involving the *MYD88* L265P mutation (9–11). However, efficacy in *MYD88* wild-type patients is uncertain (12). Preliminary exploratory therapy may highlight the pathogenic effect of underlying B-cell clone in the development of SchS and suggest a potential universal therapeutic value of clone-targeted therapy.

Moreover, even a minimal plasma-cell clone can lead to severe clinical manifestations due to the toxicity of the monoclonal immunoglobulin or other mechanisms (4). Indication for therapy in monoclonal gammopathy of clinical significance (MGCS) is driven by organ damage due to the small plasma cell clone (13). The anti-plasma cell therapy, such as bortezomib, could target both the inflammasome activation and the underlying plasma cell clone (14). To our knowledge, this case report presents the first documented use of bortezomib in SchS patients.

## Case presentation

**Figure 1:**
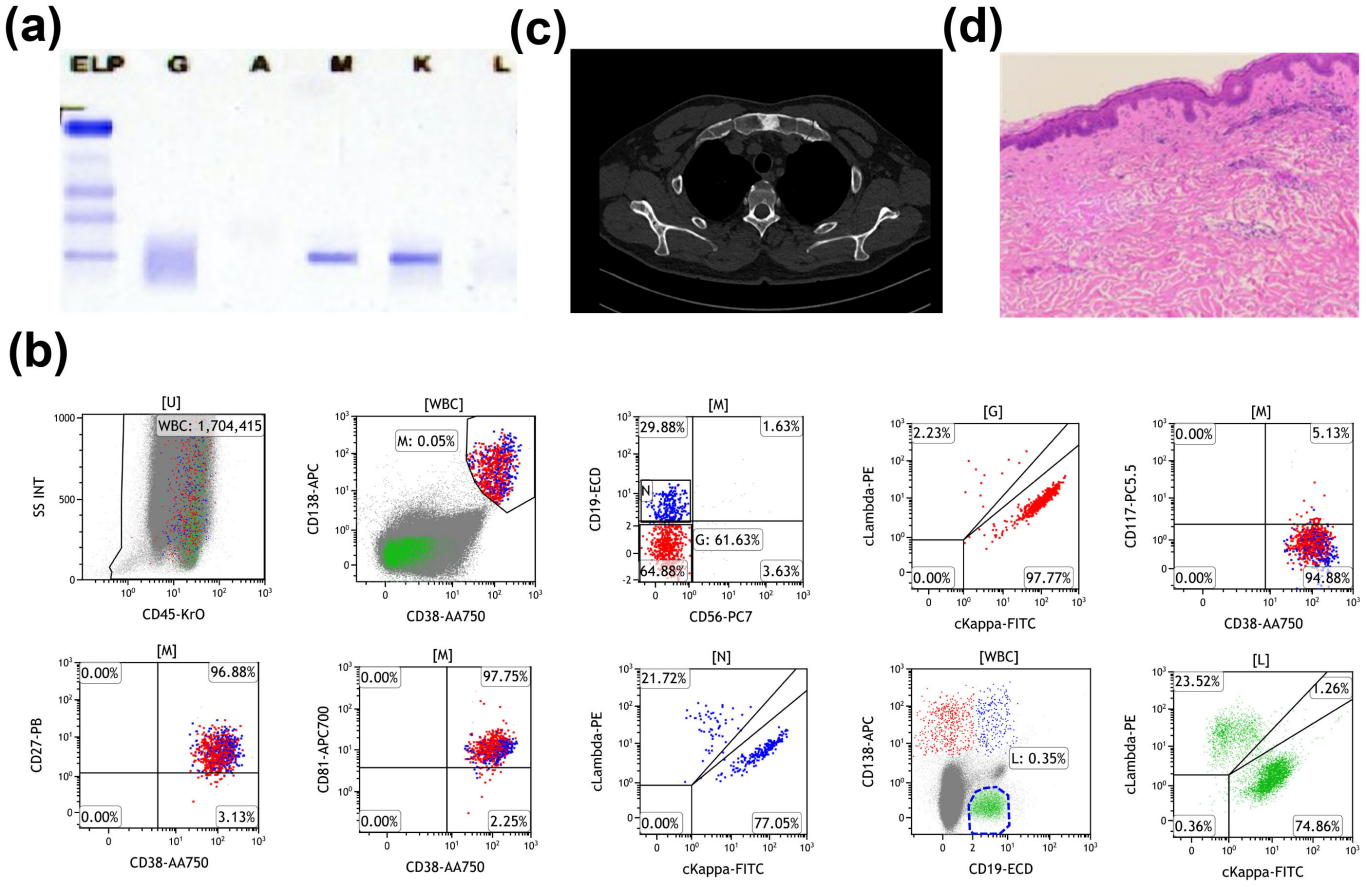
Summary of laboratory findings. **(A)** Serum protein electrophoresis before treatment. **(B)** Flow cytometry analysis of bone marrow showing 0.03% monoclonal B cells, expressing CD38, CD138, CD81, CD27, CD45, and cytoplasmic kappa light chain, with no expression of CD19, CD56, CD117, or cytoplasmic lambda light chain. **(C)** Computed tomography scans demonstrating osteosclerotic bone lesions. **(D)** Cutaneous biopsy revealing perivascular infiltration of neutrophils, consistent with neutrophilic dermatosis, without evidence of vasculitis.

A 60-year-old Chinese male was admitted to Nanjing Drum Tower Hospital in 2023 due to recurrent fever, urticarial rash, and arthralgia for 3 months. Laboratory evaluation revealed leukocytosis (10.7*10^9^/L) with neutrophilia (81.6%), elevated C-reactive protein (CRP) (43.6 mg/L), and monoclonal IgM (6.61 g/L). Serum protein electrophoresis demonstrated the presence of IgMκ monoclonal gammopathy (**Figure 1A**). Bone marrow aspiration showed no morphologic evidence of LPL, but flow cytometry identified an abnormal population of plasma cells expressing CD138, CD38, CD81, CD27, CD45, and cytoplasmic κ light chain. No abnormality was detected on lymphocyte immunophenotyping, excluding a diagnosis of LPL/WM (**Figure 1B**). Cytogenetic analysis revealed a normal male karyotype. Imaging showed osteosclerotic bone lesions (**Figure 1C**). Skin biopsy showed perivascular infiltration of neutrophils, without vasculitis change, consistent with neutrophilic dermatosis (**Figure 1D**). The combination of recurrent urticaria rash, IgMκ gammopathy, elevated CRP level, neutrophilic dermatosis without vasculitis, osteosclerotic bone lesions, and fever met two major and four minor criteria of the Strasbourg criteria for the diagnosis of SchS (15).

**Figure 2:**
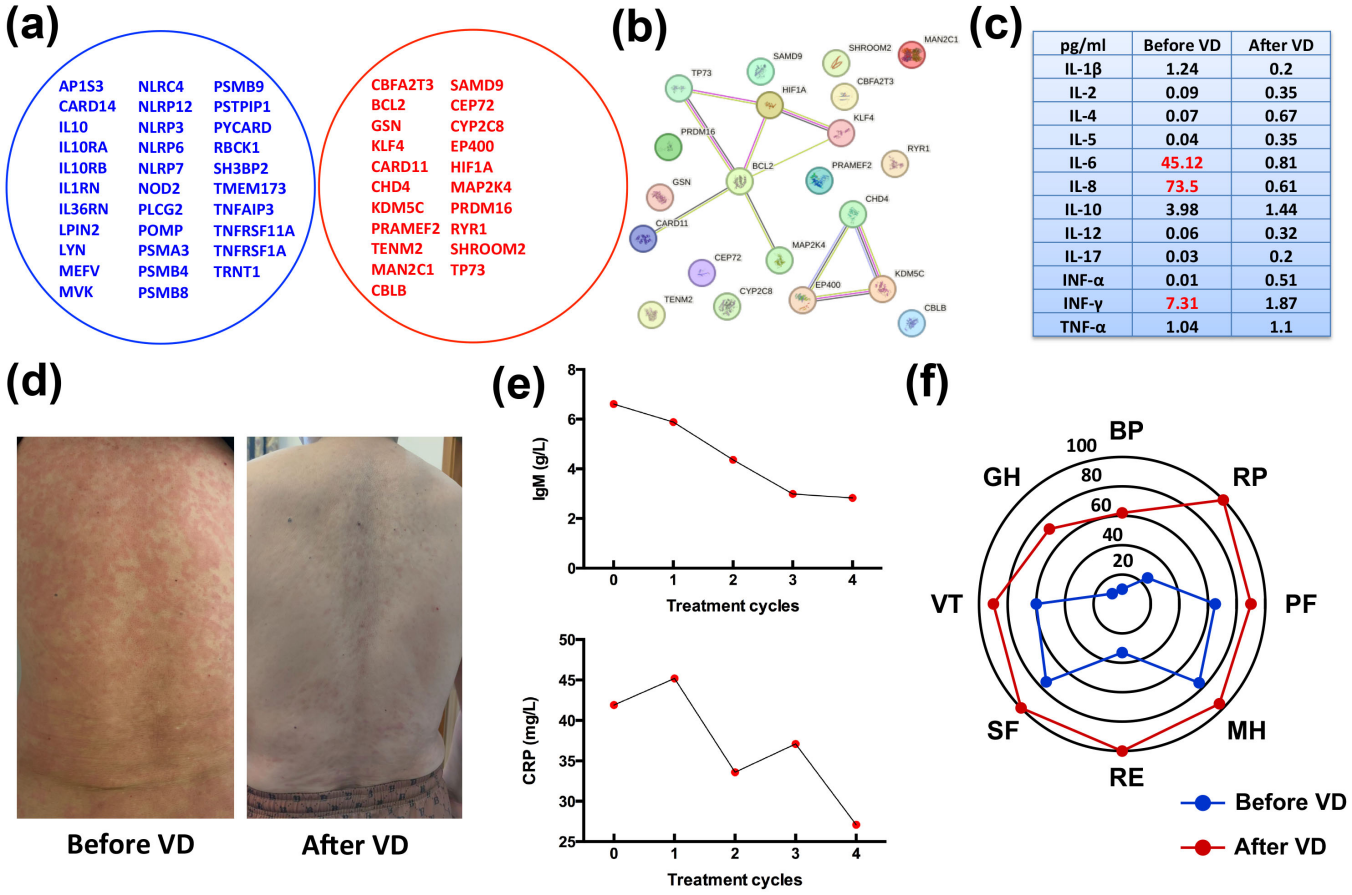
Clinical manifestations and laboratory findings after four cycles of VD treatment. **(A)** Venn diagram illustrating no overlap between 32 autoinflammatory genes and the patient’s gene mutations. **(B)** Protein interaction network constructed using the STRING online tool, with GO enrichment analysis identifying associations of aberrant genes (*PRDM16*, *KLF4*, *TP73*, *BCL2*, *HIF1A*, and *CHD4*) with DNA-binding transcription factor and NF-κB binding activity. **(C)** Inflammatory cytokine levels at different therapeutic time points. Initial levels of IL-6, IL-8 and INF-γ were elevated at diagnosis, but returned to normal after four cycles of VD treatment. **(D)** Clinical presentation of the patient before and after four treatment cycles. **(E)** Serum CRP and IgM levels showing significant reductions after treatment. **(F)** Quality of life (QoL) survey results before starting VD and after four treatment cycles. A statistically significant improvement in all domains was observed (a change of 10 points or more is considered clinically significant). BP, bodily pain; RP, role physical; PF, physical function; MH, mental health; RE, role emotional; SF, social function; VT, vitality; GH, general health.

Clinically, SchS closely mimics cryopyrin-associated periodic syndromes (CAPS), which are due to gain-of-function mutations in the *NLRP3* gene, critical for NLRP3-inflammasome activity and IL-1β production (16). The NLRP3 inflammasome plays vital effects in the innate immune system in response to a variety of stimuli. Upon the formation of the NLRP3 inflammasome, caspase-1 is released and activates the pro-inflammatory cytokines IL-1β and IL-18 (17). However, previous studies do not support a role for somatic *NLRP3* mosaicism in SchS pathogenesis (5). To identify the two diseases, whole exome sequencing (*WES)* was performed to search for somatic *NLRP3* mutations and 32 genes linked to inherited autoinflammatory diseases, with no pathological variants identified. Analysis of the 32 genes showed no mutations in *TNFRSF1A*, *NOD2*, or *NLRC4* (genes associated with dominant autoinflammatory diseases with rash) and did not identify common susceptibility factors for SchS (**Figure 2A**) (5). Gene Ontology (GO) enrichment analysis of aberrant genes (*PRDM16*, *KLF4*, *TP73*, *BCL2*, *HIF1A*, and *CHD4*) indicated associations with DNA-binding transcription factor and NF-κB binding activity (**Figure 2B**). In addition, to distinguish SchS from lymphoproliferative disorders, which often feature monoclonal gammopathy (2), *MYD88* mutations were sought, predicting the development of WM (18), and the results were negative. Now VEXAS syndrome, a newly discovered syndrome driven by somatic mutations in *UBA1*, has been identified as a link between autoinflammatory and malignant hematologic diseases (19). In order to distinguish between SchS and VEXAS, we further tested for *UBA1* mutation, and the result indicated negative (**Figure 2A**).

Cytokine profiling was performed to assess immune responses and guide treatment choices. At disease onset, the levels of IL-6, IL-8, and INF-γ were elevated, with 45.12 pg/ml, 73.5 pg/ml, and 7.31 pg/ml, respectively, as indicated in **Figure 2C**. Due to the IL-1β was not detected and limited access to IL-1 antagonist agents (anakinra or canakinumab) in China, IL-1 blockade therapies were not pursued (20–22). The patient also declined IL-6 blockade therapy with tocilizumab for financial reasons. Recently, SchS has been incorporated into the spectrum of MGCS with a characterized small plasma cell clone (13). Therefore, this patient underwent treatment with the bortezomib-dexamethasone (VD) regimen, consisting of bortezomib (1.3 mg/m^2^) on days 1, 4, 8, and 11, and dexamethasone (20 mg) on days 1, 2, 4, 5, 8, 9, 11, and 12, aiming for anti-plasma cell therapy. Following four cycles of treatment, the patient’s urticarial rash resolved completely (**Figure 2D**), and serum levels of lgM and CRP showed marked declines (**Figure 2E**), and there were moderate declines in the levels of serum CRP (27.1 mg/L) and IgM (2.83 g/L). These declines of IL-6 and CRP correspond to the patient’s improving condition (**Figure 2C**). At the four-month follow-up, the patient’s responses were accompanied by improved quality of life (QoL) (**Figure 2F**). The patient was closely monitored for side effects during VD regimen, and no infectious complications and peripheral neuropathy occurred. Follow-up consisted of monthly visits for the first four months, followed by bi-monthly assessments, which included comprehensive clinical evaluations and laboratory tests for CRP and IgM levels. These assessments were crucial in monitoring the patient's response to treatment and overall disease activity. The stable values of CRP and IgM during the follow-up period indicate a sustained response to the bortezomib therapy, suggesting effective disease control and a positive impact on the patient's clinical status. As of the last follow-up, the patient continues on bortezomib therapy, with a dosing adjustment to 1.3 mg/m^2^ monthly, reflecting a good response and tolerability to the treatment.

## Discussion

The pathophysiology of SchS remains enigmatic, with evidence indicating it involves an aberrant autoinflammatory response characterized by inflammatory cytokines such as IL-1β, IL-6, and TNF-α (23). It is hypothesized that IL-1β may trigger IL-18, facilitating the differentiation of B-lymphocytes into plasma cells and leading to monoclonal gammopathy (24). IL-18, another cytokine of the IL-1 family that is cleaved by caspase-1, which cleaves pro-IL-1β and triggers the formation of gasdermin D pores. Gasdermin D pores allow for the secretion of active IL-1β and IL-18 initiating the organism-wide inflammatory response (25, 26). The different conventional therapeutic approaches (interferon, corticosteroids, anti-rheumatic medications, and immunosuppressants) get highly variable responses, often partial and temporary (27, 28). SchS is recognized as a late-onset acquired autoinflammatory syndrome with a significant inflammatory component. Central to its pathogenesis are the proinflammatory cytokines IL-1 and IL-6, with IL-1 being particularly crucial in the syndrome's development (29, 30). Given the established role of IL-1 in the pathogenesis of the auto-inflammatory component, IL-1 blockade therapies (anakinra and canakinumab) are recommended as the first-line treatments for SchS. These treatments effectively manage symptoms and reduce inflammation in most SchS patients, but they are not disease-modifying, and discontinuation typically results in relapse (4). Moreover, IL-1 blockade dose not alter the levels of monoclonal proteins, and IgM MGUS may progress during treatment (31). The connection between IgM monoclonal gammopathy and inflammasome activation in SchS is not yet established, necessitating the exploration of treatments that can address both issues simultaneously.

The prognosis of SchS is influenced by the development of lymphoproliferative complications associated with dysglobulinemia component, such as IgM MGUS, lymphoma, and WM (32, 33). IgM monoclonal gammopathy is a defining feature of SchS according to the Strasbourg criteria. If the cell clone is lymphoplasmacytic or corresponds to chronic lymphocytic leukemia or B cell lymphoma, treatment should be adjusted accordingly, often involving an anti-CD20 monoclonal antibody (34). However, in cases where the clone does not match these conditions, such as this patient discussed, an anti-CD20 monoclonal antibody is not indicated (35). Genetic testing has revealed the presence of the *MYD88* L265P mutation in peripheral blood samples of 30% of SchS patients (36). The link between MYD88 and BTK lies in their involvement in the signaling pathways that lead to B cell activation and the production of immunoglobulins. In certain B cell malignancies and lymphoproliferative disorders, the *MYD88* L265P mutation leads to constitutive activation of the NF-κB pathway, resulting in uncontrolled B cell proliferation and survival (37, 38). This mutation is also associated with the production of monoclonal gammopathy, which can be a feature of SchS. BTK inhibitors, such as ibrutinib, target the BCR signaling pathway, which is downstream of MYD88 in the B cell activation process (39, 40). Of note is that several SchS patients have been successfully treated with the BTK inhibitor ibrutinib, which may target both IgM monoclonal gammopathy and the NLRP3 inflammasome (11, 41, 42). Compared to the successful ibrutinib-treated cases, our patient had no *MYD88* L265P mutation (7). It also highlights the importance of exploring clone-directed therapy. Above observations support the inclusion of SchS within the spectrum of MGCS, where organ damage is related to monoclonal protein or other paraneoplastic mechanisms (13).

The cytokine profiles of this patient align with our treatment options. The direct activation of IL-6 production by IL-1β, as well as the direct production of IL-6 by the NF-κB signaling, accounts for the involvement of these two cytokine pathways in the increase of CRP (43–45). Indeed, elevated IL-6 and CRP account for constitutional symptoms of SchS. Furthermore, the GO analysis has shown the functional enrichments (DNA-binding transcription factor and NF-κB binding activity) in this patient’s network (46). As we all know, NF-κB regulates the transcription of numerous genes involved in immune response, cell apoptosis, differentiation, proliferation, adhesion, and angiogenesis (47, 48). The expression of IL-1, IL-6, and CRP is mediated by activation of NF-κB signaling pathways (43, 49). Multiple kinds of NF-κB pathway mutations have been identified in various inflammatory diseases, lymphomas, and MM. Even a small plasma cell clone can produce severe clinical manifestations due to toxicity of the monoclonal immunoglobulin. To enhance early diagnosis and management of these small plasma cell clone-related disorders, the concept of MGCS has been proposed (13). Treatment in MGCS is driven by organ damage caused by the secreting plasma clone. The current treatment strategy primarily relies on repeated courses of bortezomib combined with dexamethasone, which produces rapid and deep hematological responses in various MGCSs (14) [POEMS syndrome (50), Light chain deposition disease (51), TEMPI (52), Cryoglobulinemia (53), Scleromyxedema (54), and Clarkson disease (55)]. Bortezomib, a proteasome inhibitor (PI) targeting at the ubiquitin-proteasome pathway, is the first clinically approved PI for the treatment of MM (56). The ubiquitin-proteasome system (UPS) is recognized as the major pathway for degradation of intracellular proteins, many of which play an important role in the regulation of pro-inflammatory cytokines. UPS deregulation is involved in several pathological processes such as malignant hematologic diseases, viral infections, and autoimmunities (57, 58). Bortezomib was demonstrated to be a rapid and potent inhibitor of NF-κB-inducible cytokines, including TNF-α, IL-1β, IL-6, and IL-10 (59). Hence, it was supposed that bortezomib can inhibit NF-κB activation process and indirectly produces a potential anti-inflammatory response for treating SchS. It is known that glucocorticosteroid treatment could result in diminution of symptoms and normalization of the cytokine responses in SchS patients (2). It should be noted that this patient was started on prednisone 30 mg daily by an immunologist. However, his clinical symptoms did not improve, and the rash got worse. Above all, the direct or indirect NF-κB inhibitory effects of VD regimen reflect the effectiveness of this combined treatment on both entities, regardless of the potential trigger role of one on the other. Bortezomib represents a novel and unique therapeutic option for SchS patients, targeting both the underlying IgM monoclonal gammopathy and inflammasome activation. While it is recognized that even minimal plasma cell clones can lead to severe clinical manifestations due to the potential toxicity of monoclonal immunoglobulins or other mechanisms, the precise role of these clones and IgM paraprotein secretion in the pathogenesis of SchS remains a subject of investigation (60). Some researchers posit that the presence of such clones may be an epiphenomenon, not directly contributing to the disease's core pathophysiology (61, 62). However, others suggest that they may play a more active role, possibly influencing the inflammatory process (63–65). The variability in clinical presentations and responses to treatment among SchS patients supports the need for further exploration into the significance of these clones and the potential mechanisms by which they might contribute to the disease. Future studies are warranted to clarify the relationship between minimal plasma cell clones, IgM paraprotein secretion, and the development of SchS.

## Conclusion

In conclusion, while treatments for SchS have been reported with anti-IL-1 therapies and BTK inhibitors, there is no report on anti-plasma cell therapies. The successful remission of clinical symptoms, alongside a decrease in IgM monoclonal protein and improvement in quality of life, suggests that anti-plasma cell therapies such as bortezomib should be considered in the treatment of SchS.

## Data Availability

The raw data supporting the conclusions of this article will be made available by the authors, without undue reservation.
